# Trends in warfarin use and its associations with thromboembolic and bleeding rates in a population with atrial fibrillation between 1996 and 2011

**DOI:** 10.1371/journal.pone.0194295

**Published:** 2018-03-16

**Authors:** Peter Wæde Hansen, Thomas S. G. Sehested, Emil Loldrup Fosbøl, Christian Torp-Pedersen, Lars Køber, Charlotte Andersson, Gunnar H. Gislason

**Affiliations:** 1 The Danish Heart Foundation, Copenhagen, Denmark; 2 The Heart Centre, Rigshospitalet, University of Copenhagen, Copenhagen, Denmark; 3 Institute of Health, Science and Technology, Aalborg University, Aalborg, Denmark; 4 The National Institute of Public Health, University of Southern Denmark, Copenhagen, Denmark; 5 Department of Clinical Medicine, University of Copenhagen, Copenhagen, Denmark; Inselspital Universitatsspital Bern, SWITZERLAND

## Abstract

**Aim:**

Warfarin is a cornerstone for the prevention of thromboembolism in atrial fibrillation (AF), and several efforts have been taken to increase its usage and safety, including risk stratification schemes. Our aim was to investigate the temporal trends in initiation of warfarin and its effects on incidence of bleeding and thromboembolism in patients with new-onset atrial fibrillation 1996–2011.

**Methods:**

All patients with a first-time diagnosis of non-valvular atrial fibrillation were identified from nationwide administrative registries. Trends were determined by linear regression.

**Results:**

In total 153,682 patients were included. Initiation of warfarin increased from 14% to 41% (p<0.0001). Events of thromboembolism decreased from 3.9% to 2.6% annually (p<0.0001). The greatest decline in thromboembolic events was observed for patients with a CHA_2_DS_2_VASc score >1, where the annual decline was -0.12% (95%CI: -0.161; -0.084)) for those treated with warfarin and -0.073% (95%CI: -0.116;-0.030)) for those not treated with warfarin. Bleeding increased from 3.3% to 3.9% (p = 0.043). For those with a CHA_2_DS_2_VASc score >1 annual bleeding rates increased by 0.095% (95%CI: -0.025; -0.165) in warfarin treated and by 0.056% (95%CI: -0.013; -0.100) in patients not treated with warfarin.

**Conclusion:**

Warfarin use increased by nearly a 3-fold between 1996 and 2011. During the same period, thromboembolic events declined by a third and bleeding increased by a fifth, suggesting a beneficial effect associated with higher warfarin use. Notably, a small decline in thromboembolic events and increase in bleeding events was observed for the untreated population, suggesting a changing risk profile of AF patients.

## Introduction

Over the past couple of decades, atrial fibrillation (AF) has been increasingly recognized as a chronic condition associated with significant risks of thromboembolism and mortality [1]. AF is also one of the major illnesses of the elderly generation. At the age of 40 years, the lifetime risk of developing AF is estimated to be 1 in 4 [2]. Warfarin has been a cornerstone in the preventive treatment of AF-associated stroke and several risk schemes have been developed in the last decade (e.g., CHADS_2_ score, CHA_2_DS_2_VASc score, HAS-BLED score), which has facilitated treatment decisions. In addition, guidelines have been gradually changed, now recommending treatment for patients at lower risk, but it is unclear how this has affected the prevention of thromboembolism and the serious side effect of bleeding.

Population-based studies investigating the proportion and characteristics of AF patients treated with warfarin and the temporal trends in thromboembolism and bleeding are sparse. Therefore, we investigate whether 1) the proportion of incident AF patients initiating warfarin over the years has increased and 2) if the temporal trends in incidence of bleeding and thromboembolism have changed.

## Methods

The study was based on the Danish administrative health care registries. In brief, the Danish healthcare system is tax financed and without copayment. To keep track of expenses and for quality purposes, nationwide healthcare related registries have been developed since the 1970s. At birth or immigration, all citizens are given a unique identity number, which is used in all contacts with the hospital system. For the present analyses, the identity number was used to cross-link four registries containing medical information about all Danish inhabitants (nearly 6 million people). Information on gender, date of birth and death were collected from The National Population Registry [3]. Hospitalization was registered in The Danish National Patient Registry currently using the 10^th^ version of The International Classification of Diseases (ICD-10). Hospital departments are reimbursed based on accurate diagnostic and procedural registration, why this information is very reliable [4]. Information about filled prescriptions were retrieved from The Danish Registry of Medicinal Product Statistics [5] using The AnatomicalChemical Therapeutical [ATC] classification system. This registry monitors the use of drugs and contains information about all dispensed prescriptions since 1995. It is an electronic dispensing system considered accurate and complete. Causes of death were found in the Danish Register of Causes of Death[6]. This registry is currently based on the ICD-10 and the reliability depends mainly on the physicians notification.

### Study population and observational period

We identified patients hospitalized with non-valvular atrial fibrillation (S1 Definitions) for the first time between 1996 and 2011.Patients younger than 30 years of age or with a previous diagnosis of AF (1978–1995) were excluded. We anticipated that patients younger than 30 years of age could have developed atrial fibrillation secondary to other causes, including e.g. congenital heart disease. The positive predictive value of the diagnosis of atrial fibrillation (ICD-8 codes 427.93 and 427.94; ICD-10 code I48) is 99% in the Danish National Patient Registry [7].

The population had a 30 days grace period after being discharged from the hospital with a first time diagnosis of AF. Patients who died within the grace period were not included in the analyses to allow all patients equal time to claim a prescription. The index date was defined as the first day after the grace period. Patients were followed for one year. ‘Year’ is throughout the text defined as the year of the first AF diagnosis. An event occuring in 1997 in a patient with an AF diagnosis from 1996 will be showed as the year 1996. Events were based on The Danish National Patient Registry and Danish Register of Causes of Death covering deaths, in- and out-hospital patients and emergency room visits.

### Comorbidities

All comorbidities except for diabetes and hypertension were identified using the Danish National Patient Registry [8]. All codes are provided in S2 Definitions. Diabetes was identified by a claimed prescription of glucose-lowering medication (ATC A10) within 120 days to the index date. A validated algorithm based on a combination of at least two different antihypertensive agents was used as a proxy for identifying hypertension [9]. Definitions of concomitant medical therapy are provided in S3 Definitions.

### Anticoagulants and platelet inhibitors

Use of warfarin (ATC codes B01AA03) was based on prescription fillings in the grace period and 60 days prior to the AF date (ie, totally 90 days prior to the index date). Non-vitamin K antagonist oral anticoagulation was introduced in Denmark in the second half of 2011 and was not included in this study. Patients having a precription of an antithrombotic agent (ATC codes B01A) in the grace period besides warfarin, clopidogrel (ATC codes B01AC04) and aspirin (ATC codes B01AC06), were not included.

### Exposure groups and outcome

Two groups were compared: Patients using warfarin and patients not using warfarin. Both groups were allowed to use aspirin or clopidogrel. Two outcomes were defined: First time bleeding and first time thromboembolism—for diagnostic codes see S4 Definitions. A patient with a previous event of bleeding was only studied regarding events of thromboembolism (TE) and vice versa. Both fatal and non-fatal events were included. Non-fatal events required a hospital visit and were based on the National Population Registry. Fatal events were identified using the National Causes of Deaths Registry.

### Statistic

Patient characteristics are given as percentages (for discrete variables) or as medians with interquartile range (for continuous variables). Outcomes were stratified relative to the CHA_2_DS_2_-VASc score (0, 1, and >1) and specified as percentage of bleeding and TE. We stratified in relation to the CHA_2_DS_2_VASc score because it was an important instrument in the decision of anticoagulant therapy and enabled us to observe the historical impact of changes in clinical recommendations. We applied linear regression to determine the overall temporal trend, using the one-year event rate (TE or bleeding) as the dependent variable and year of AF as the independent variable. The fit of the linear regression was determined by the coefficient of determination (R^2^). The difference between year 1996 (study start) and year 2011 (end of study) was evaluated using a chi-squared test. A two sided p-value below 0.05 was considered statistically significant.

All statistical calculations were performed using SAS (version 9.4 for Windows; SAS Institute Inc, Cary, North Carolina) or R (version 3.2.3 for Windows; The R Foundation).

### Sensitivity analysis

The 30 days grace period may hide a higher event rate in the first period after an AF diagnosis. To ensure the robustness of the present findings, a sensitivity analysis applying a 7 day grace period was used. Furthermore, we examined how including patients with a prescription of another antithrombotic agent than warfarin, clopidogrel and aspirin within the grace period, affected the result.

### Ethics

The study was approved by the Danish Data Protection Agency (Ref.no. 2007-58-015 I-Suite nr: 02720, GEH-2014-012). The data were made available at the individual level in such a way that specific individuals could not be identified. In Denmark, retrospective register-based studies do not need ethical approval.

## Results

The study population contained 153,682 patients with a median age of 75.57 years (interquartile range 17.1), and 50.8% being male. The flow-chart diagram of exclusions is illustrated in S1 Fig. Baseline characteristics are presented in Table 1 with the temporal development in characteristics illustrated in S2 Fig. From 2003 to 2012 the ratio of in-patients with a first time AF diagnosis changed from 77.0% to 73.1% and the number of out-patients increased from 15.9% to 21.7%. The remaining part were emergency room patients, declining from 7.1% to 5.2%.

**Table 1:** Baseline characteristics.

		CHA2DS2VASC score = 0		CHA2DS2VASC score = 1		CHA2DS2VASC score ≥ 1	
	*Characteristic*	Warfarin	Non-warfarin	Warfarin	Non-warfarin	Warfarin	Non-warfarin
	No.	3,169	8.642	6,646	13,939	42,050	79,236
	Age—yr (median)	57.2	53.5	65.1	60.8	76.3	80.4
	age—yr (mean)	55.3 (7.2)	51.5 (9.0)	63.9 (8.0)	59.3 (10.3)	75.3 (8.7)	79.2 (9.7)
	**Risk factores for TE—No. (%)**						
C	Congestive heart failure	0 (0.0)	0 (0.0)	322 (4.8)	409 (2.9)	12,345 (29.4)	21,686 (27.4)
H	Hypertension	0 (0.0)	0 (0.0)	1,478 (22.2)	1,622 (11.6)	23,664 (56.3)	29,446 (37.2)
A_2_	> = 75 yr	0 (0.0)	0 (0.0)	0 (0.0)	0 (0.0)	23,799 (56.6)	55,892 (70.5)
D	Diabetes mellitus	0 (0.0)	0 (0.0)	155 (1.9)	258 (2.3)	4,662 (9.5)	7,516 (11.1)
S_2_	History of stroke or systemic thromboembolim	0 (0.0)	0 (0.0)	0 (0.0)	0 (0.0)	11,647 (14.7)	7,750 (18.4)
V	Vascular disease	0 (0.0)	0 (0.0)	176 (2.6)	660 (4.7)	8,022 (19.1)	18,331 (23.1)
A	65–74 yr	0 (0.0)	0 (0.0)	3,622 (54.5)	4,953 (35.5)	14,398 (34.2)	18,444 (23,3)
S_c_	Female sex	0 (0.0)	0 (0.0)	893 (13.4)	6,037 (43.3)	20,737 (49.3)	47,896 (60.4)
	**Risk factores for bleeding—No. (%)**						
H	Hypertension	0 (0.0)	0 (0.0)	1,478 (22.2)	1,622 (11.6)	23,664 (56.3)	29,446 (37.2)
A	Abnormal liver function	41 (1.29)	273 (3.16)	108 (1.63)	414 (3.0)	585 (1.39)	1560 (1.97)
	Renal disease	33 (1.04)	103 (1.19)	94 (1.4)	299 (2.15)	1,284 (3.05)	3,209 (4.05)
S	History of stroke or systemic thromboembolim	0 (0.0)	0 (0.0)	0 (0.0)	0 (0.0)	11,647 (14.7)	7,750 (18.4)
B	History of bleeding	230 (6.4)	678 (7.8)	670 (10.1)	1,569 (11.3)	4,794 (11.4)	11,987 (15.1)
E	Age > = 65 yr	0 (0.0)	0 (0.0)	3,622 (54.5)	4,953 (35.5)	38,197 (90.8)	74,336 (93.8)
D	Alcohol abuse	187 (5.9)	806 (9.3)	287 (4.3)	911 (6.5)	1,097 (2.6)	2,239 (2.8)
	Use of NSAIDs	619 (19.5)	2,077 (24.0)	1,774 (26.7)	4,646 (33.4)	16,905 (40.2)	43,592 (55.0)
	**Antithrombotic medication—No. (%)**						
	Aspirin	367 (16.7)	1,443 (11.6)	1,232 (24.0)	3,343 (18.5)	13,472 (46.1)	36,566 (32.0)
	Clopodogrel	7 (0.22)	36 (0.42)	53 (0.22)	188 (1.35)	1,208 (2.)	3,707 (4.68)
	**HAS-BLED score—No. (%)**						
	0 or 1	3,046 (96.1)	8,031 (92.9)	4,626 (69.6)	9,874 (70.8)	9,552 (22.7)	18,107 (22.9)
	2	109 (3.4)	475 (5.5)	1,715 (25.8)	3,235 (23.2)	16,788 (39.9)	31,106 (39.3)
	≥ 3	14 (0.4)	136 (1.6)	305 (4.6)	830 (6.0)	15,710 (37.4)	30,023 (37.9)
	**Medication—No. (%)**						
	Proton pump inhibitor	183 (5.8)	683 (7.9)	552 (8.3)	1631 (11.7)	1067 (9.9)	2890 (15.2)
	Digoxin	1.132 (35.7)	752 (8.7)	2,901 (43.7)	2.214 (15.9)	5,347 (49.5)	5.768 (30.3)
	Calcium channel blocker	462 (14.6)	491 (5.7)	1,532 (23.1)	1,850 (13.3)	2,750 (25.5)	3,505 (18.4)
	Beta blocker	1,660 (52.4)	2,735 (31.6)	3,581 (53.9)	5,304 (38.1)	5,747 (53.2)	6,716 (35.3)
	Statins	221 (7.0)	453 (5.2)	812 (12.2)	1,410 (10.1)	1,690 (15.6)	2,256 (11.9)
	Amiodarone	87 (2.7)	88 (1.0)	219 (3.3)	269 (1.9)	405 (3.7)	511 (2.7)
	Antiarrhythmic	80 (2.5)	177 (2.1)	106 (1.6)	252 (1.8)	117 (1.1)	251 (1.3)

The first column indicates the letter of the variables in the CHA_2_DS_2_VASc or HAS-BLED score. As stated in limitations, we do not have information about Labile INR in the HAS-BLED score why ‘L’ is not included.

### Temporal warfarin use

**Fig 1:**
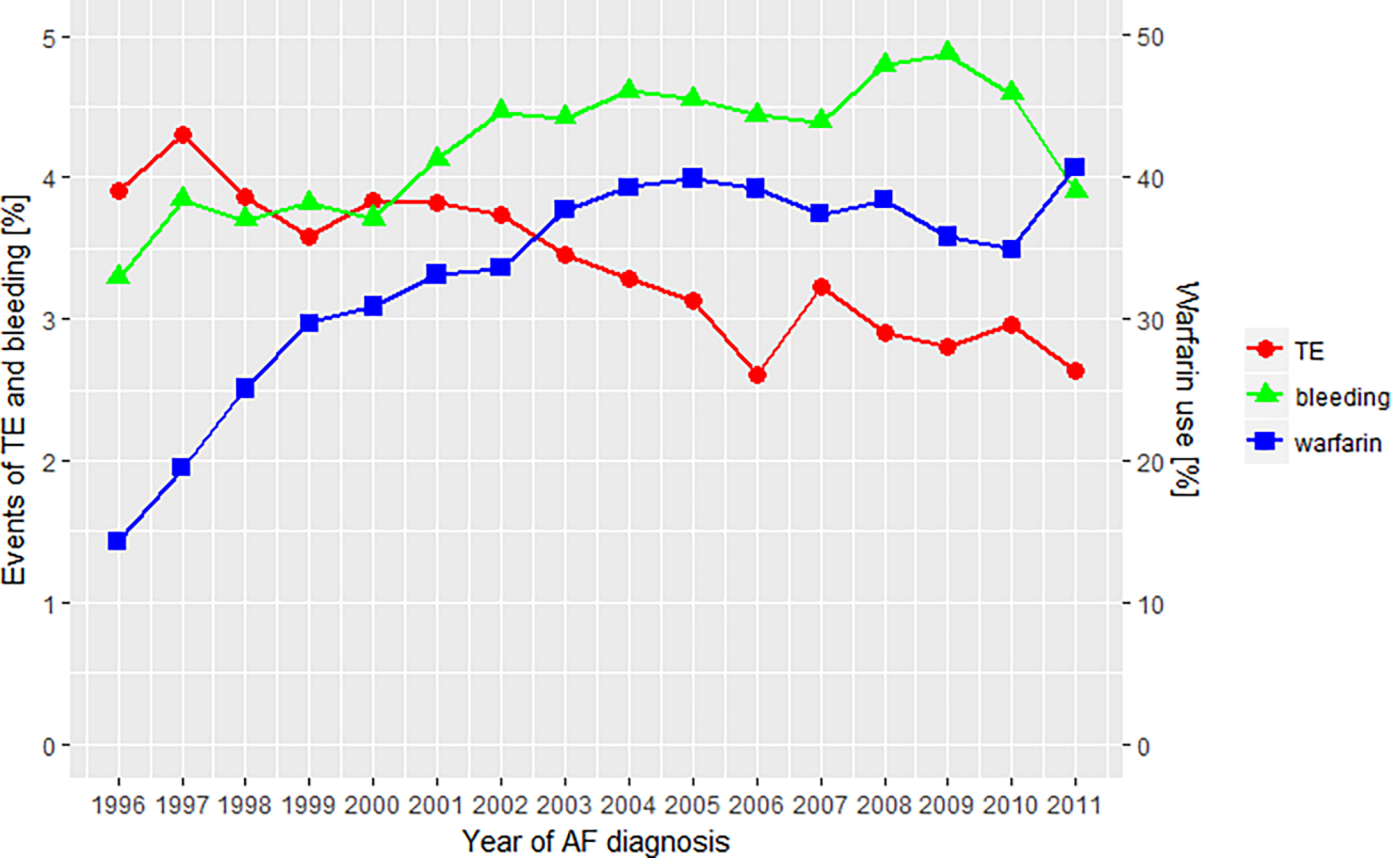
Temporal trends of TE, bleeding and warfarin. Left axis illustrates the percentage of AF patients having an event within the first year. Right axis illustrates the percentage of patients initiating warfarin. TE indicates events of thromboembolism; bleeding, events of bleedings; warfarin, initiation of warfarin; and Year of AF diagnosis, the year the patients got their AF diagnosis.

**Fig 2:**
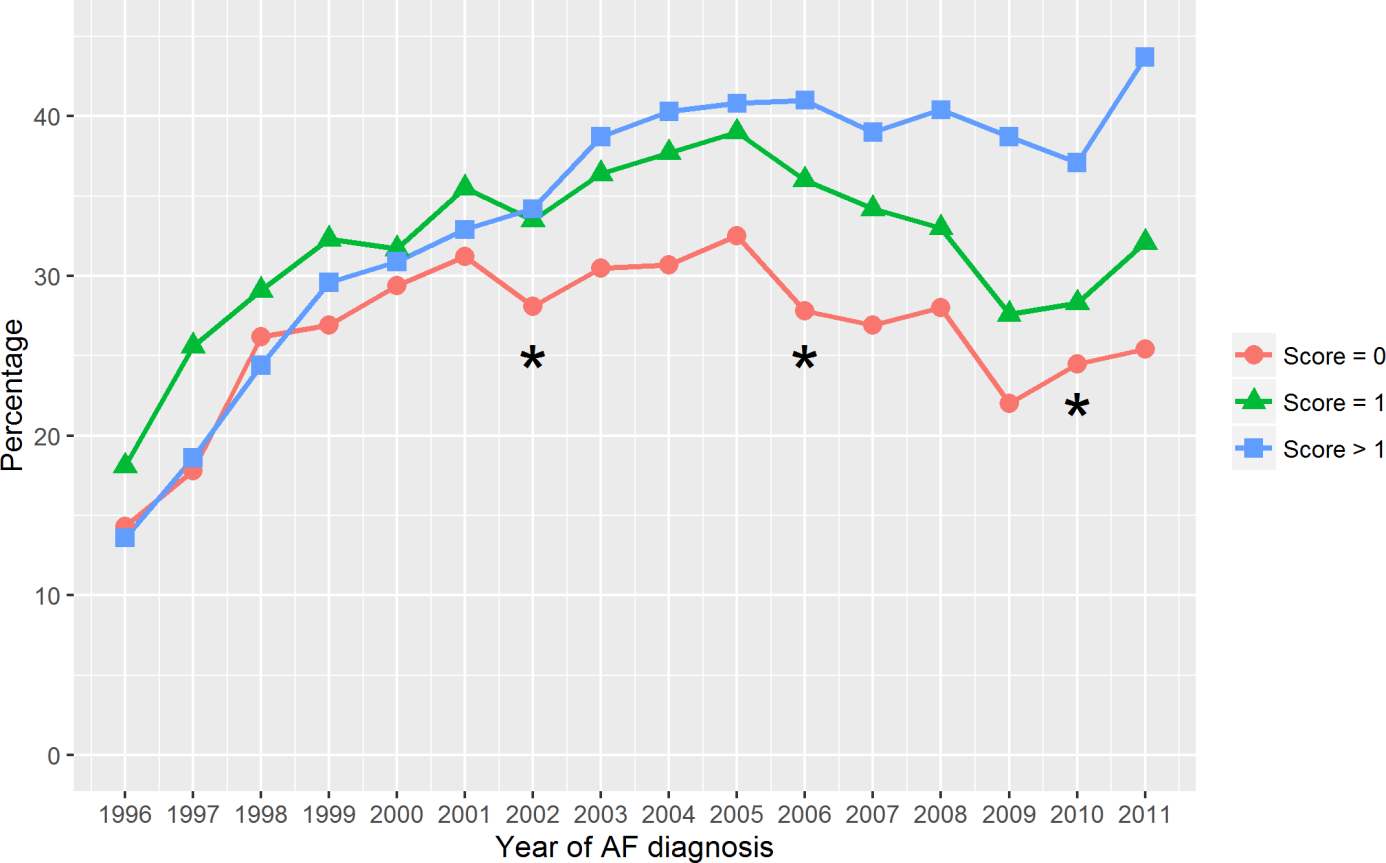
Procentage of patients in warfarin treatment. Warfarin application relative to the CHA_2_DS_2_VASc score. Score = 0 indicates a CHA_2_DS_2_VASc score of 0; Score = 1, a CHA_2_DS_2_VASc score of 1; Score > 1, a CHA_2_DS_2_VASc score above 1 and Year of AF diagnosis, the year the patients got their AF diagnosis. ‘*’, New guidelines.

The proportion of incident AF patients claiming a prescription of warfarin increased from 14% in 1996 to 41% in 2011 (p-value for difference < 0.0001), Fig 1. Stratifying relative to the CHA_2_DS_2_-VASc score (0, 1, and >1), all groups increased proportionally until 2001– Fig 2. Between 2001 and 2005 the proportion of patients in warfarin therapy having a CHA_2_DS_2_-VASc -score of 0 leveled of, while it increased in patients with a CHA_2_DS_2_-VASc -score above 0. After 2005 a decline was observed in patients with a CHA_2_DS_2_-VASc score of 1 or below and leveled of in patients with a CHA_2_DS_2_-VASc score above 1. In 2011 the proportion increased again, particularly in patients having a CHA_2_DS_2_-VASc score above 0.

### Thromboembolism

**Fig 3:**
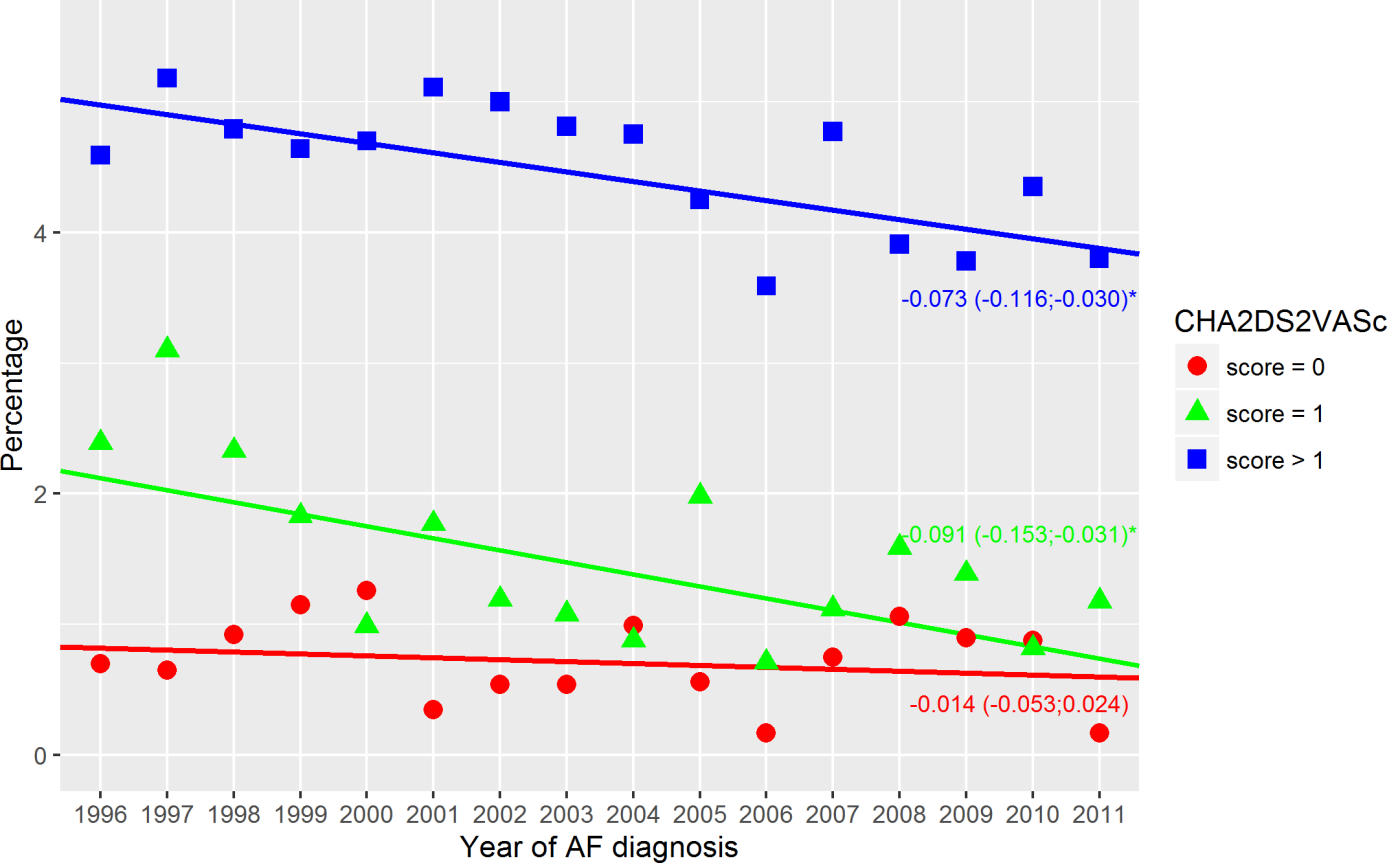
Thromboembolic events–non-warfarin treated. Thromboembolism within the first year in non-warfarin treated patients with a CHA2DS2VASc score of 0, 1 or above 1. Dots indicate the crude event rate. The slopes are specified with the 95% confidence interval in brackets. R^2^ were: 0.05; 0.43; 0.49 (score = 0; score = 1; score > 1). Score = 0 indicate a CHA_2_DS_2_VASc score of 0; Score = 1, a CHA_2_DS_2_VASc score of 1; Score > 1, a CHA_2_DS_2_VASc score above 1; and Year of AF diagnosis, the year the patients got their AF diagnosis. ‘*’ A significant slope (a two side p-value below 0.05).

**Fig 4:**
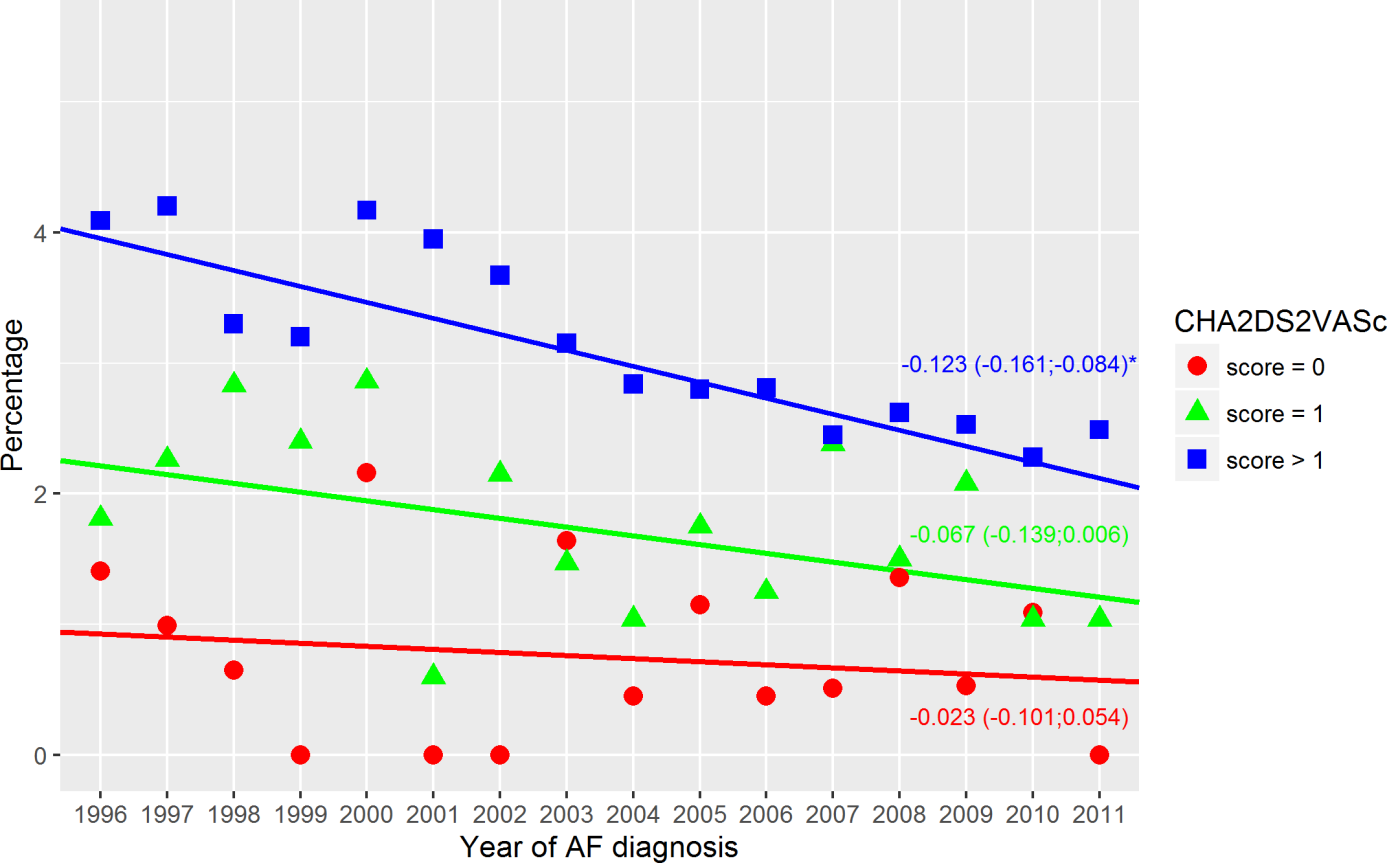
Thromboembolic events–warfarin treated. Thromboembolism within the first year in warfarin treated patients stratified relative to the CHA_2_DS_2_VASc score. Dots indicate the crude event rate. The slopes are specified with the 95% confidence interval in brackets. R^2^ were: 0.03; 0.22; 0.77 (score = 0; score = 1; score > 1). Score = 0 indicate a CHA_2_DS_2_VASc score of 0; Score = 1, a CHA_2_DS_2_VASc score of 1; Score > 1, a CHA_2_DS_2_VASc score above 1; and Year of AF diagnosis, the year the patients got their AF diagnosis. ‘*’ A significant slope (a two side p-value below 0.05).

The crude event rate of TE in the first year of AF declined from 3.9% in 1996 to 2.6% in 2011 (p-value for difference < 0.0001), Fig 1. In patients with a CHA_2_DS_2_-VASc score above 1, a significant decline was found in both the warfarin and non-warfarin treated patients–Figs 3 and 4. The warfarin treated patients with a CHA_2_DS_2_-VASc score above 1 had a decline of -0.123%/year (95%CI: -161; -0.084) changing from 4.1% in 1996 to 2.5% in 2011 (p-value for difference = 0.027). The non-warfarin treated patients with a CHA_2_DS_2_-VASc score above 1 had the highest event rate and, compared to warfarin treated patients, a less prominent decline of -0.073%/year (95%CI: -0.116;-0.030) changing from 4.6% in 1996 to 3.8% in 2011 (p-value for difference = 0.071).

### Bleeding

**Fig 5:**
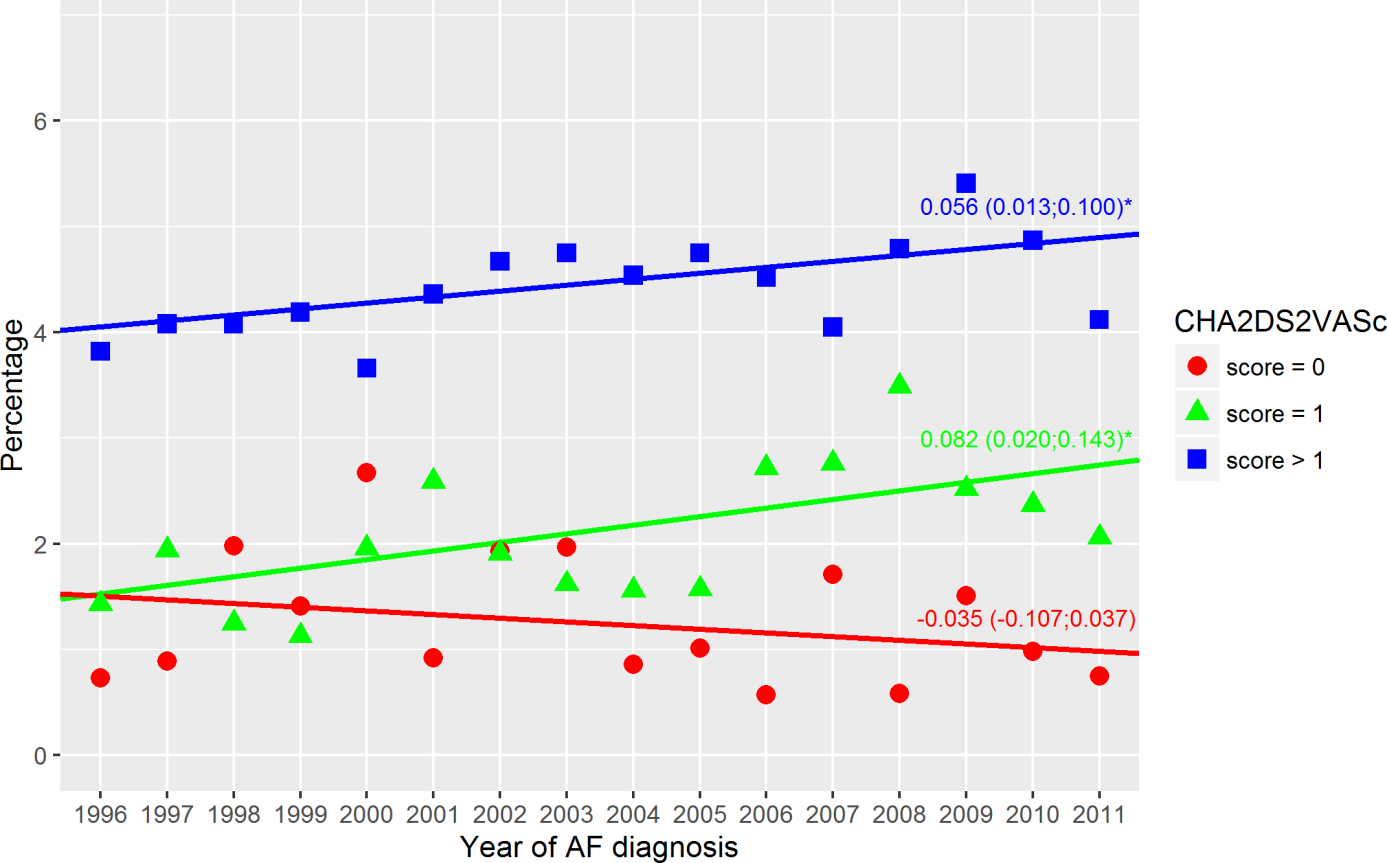
Bleeding events–non-warfarin treated. Bleeding within the first year in non-warfarin treated patients stratified relative to the CHA_2_DS_2_VASc score. Dots indicate the crude event rate. The slopes are specified with the 95% confidence interval in brackets. R^2^ were: 0.00; 0.32; 0.31 (score = 0; score = 1; score > 1). Score = 0 indicate a CHA_2_DS_2_VASc score of 0; Score = 1, a CHA_2_DS_2_VASc score of 1; Score > 1, a CHA_2_DS_2_VASc score above 1; and Year of AF diagnosis, the year the patients got their AF diagnosis. ‘*’ A significant slope (a two side p-value below 0.05).

**Fig 6:**
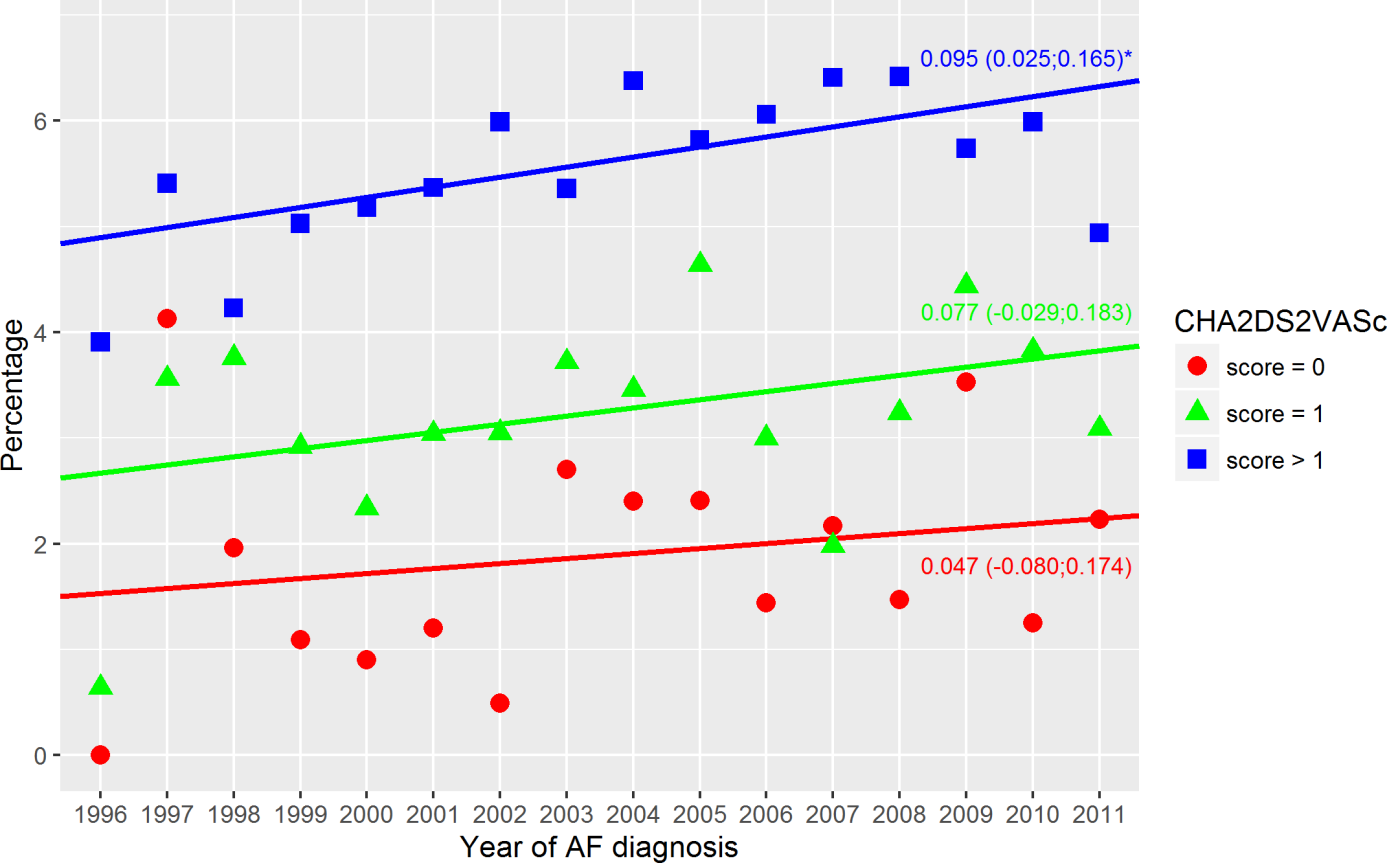
Bleeding events–warfarin treated. Bleeding within the first year in warfarin treated patients stratified relative to the CHA_2_DS_2_VASc score. Dots indicate the crude event rate. The slopes are specified with the 95% confidence interval in brackets. R^2^ were: 0.04; 0.15; 0.37 (score = 0; score = 1; score > 1). Score = 0 indicate a CHA_2_DS_2_VASc score of 0; Score = 1, a CHA_2_DS_2_VASc score of 1; Score > 1, a CHA_2_DS_2_VASc score above 1; and Year of AF diagnosis, the year the patients got their AF diagnosis. ‘*’ A significant slope (a two side p-value below 0.05).

**Fig 7:**
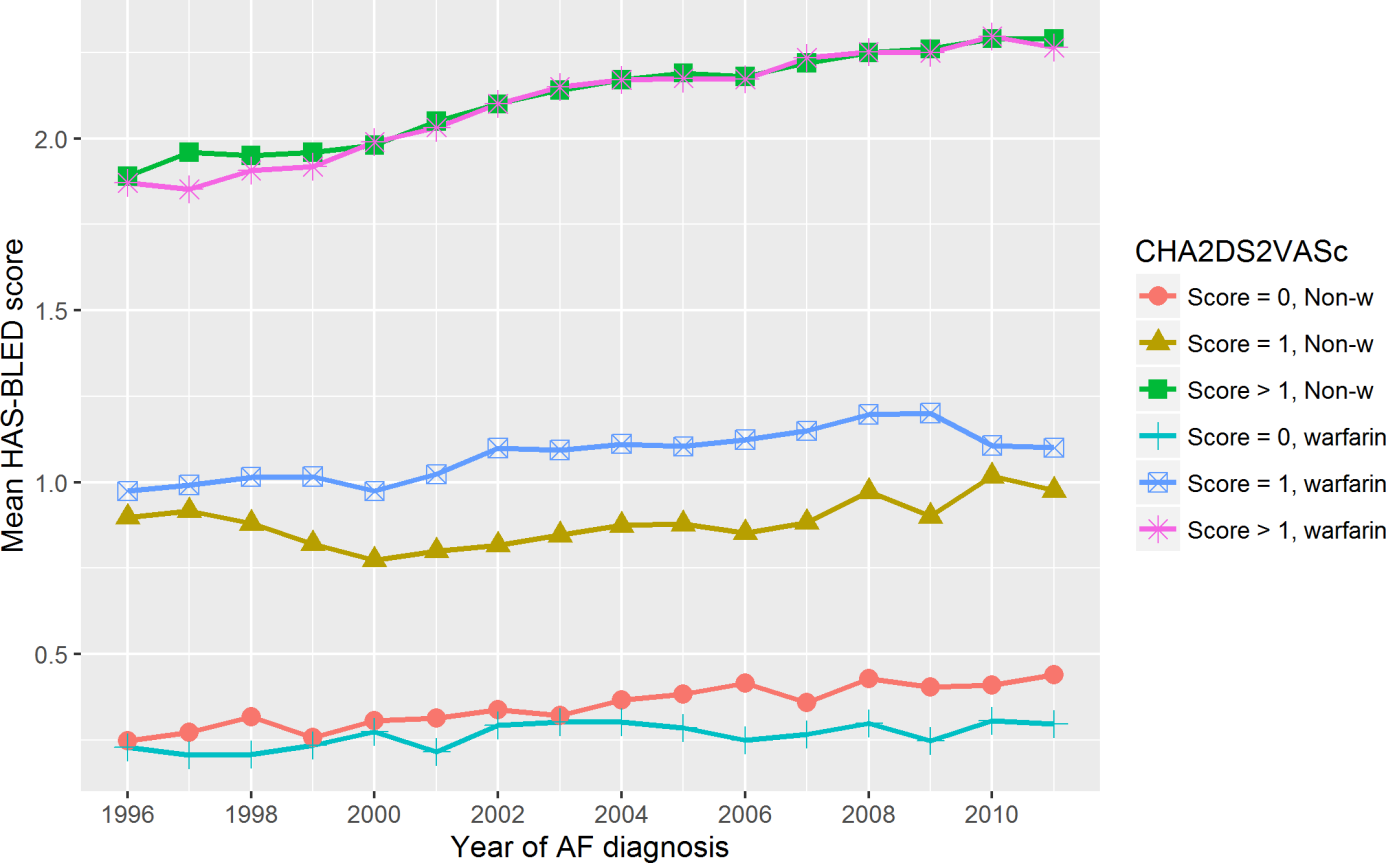
The HAS-BLED score relative to the CHA_2_DS_2_VASc score. The HAS-BLED score stratified relative to the CHA_2_DS_2_VASc score. We did not have information of thrombin time. Score = 0 indicate a CHA_2_DS_2_VASc score of 0; Score = 1, a CHA_2_DS_2_VASc score of 1; Score > 1, a CHA_2_DS_2_VASc score above 1; and Year of AF diagnosis, the year the patients got their AF diagnosis.

The crude event rate of bleeding in the first year of AF increased from 3.2% in 1996 to 3.9% in 2011 (p-value for difference = 0.043), Fig 1. The warfarin treated patients with a CHA_2_DS_2_-VASc score above 1 had the highest rate changing from 3.9% in 1996 to 4.9% in 2011 (p-value for difference = 0.230), Figs 5 and 6. These patients (warfarin treated with a CHA_2_DS_2_-VASc score above 1) had an increased mean HAS-BLED score changing from 1.87 in 1996 to 2.26 in 2011 (p-value for difference < 0.001), Fig 7.

### Sensitivity analyses

The results were mainly unaffected when applying a 7 days grace period (instead of a 30 days grace period), S3–S5 Figs. Similarly, when not excluding patients with a prescription of another antithrombotic agent than warfarin, clopidogrel and aspirin in the grace period the results were robust, S6 Fig.

## Discussion

Between 1996 and 2011 a 3-fold increase in warfarin treatment was observed among incident AF patients (14% in 1996 to 41% in 2011). This was associated with a positive effect on the prevention of TE (3.90% in 1996 to 2.63% in 2011). We observed a declining trend in both the treated and non-warfarin treated patients with the greatest decline in the treated patients with a CHA_2_DS_2_VASc score above 1 (-0.123%/year). The incidence of bleeding–a side effect of warfarin therapy–increased together with the greater use of warfarin (3.29% in 1996 to 3.90% in 2011). Of note, patients without use of warfarin had a lower rate of TE and higher rate of bleeding over the years, suggesting a changing risk profile among AF patients over the year. Over time, more patients with a first time diagnosis of AF changed from in-patients to out-patients.

### Warfarin

The proportion of incident AF patients using warfarin increased over time. This may be due to an increased awareness of the hazard of untreated AF [10]. The guidelines from 2001 [11] recommended warfarin to all patients above sixty years and patients with heart failure. This may explain why patients with a CHA_2_DS_2_VASc score of 0 was detached, Fig 2. From August 2006 [12] a single moderate risk factor (for instance age above 75 years) did not necessarily trigger warfarin therapy (aspirin was an alternative), S1 Table. Female gender, vascular disease, and age below 75 years were not classified as important risk factors. Therefore, a CHA_2_DS_2_VASc score of 4 was obtainable without warfarin been recommended. This may explain the decline of all strata and particularly the ones having low scores. In 2011 a sudden increase was observed, particularly in patients with a CHA_2_DS_2_VASc score above 0. The guidelines from August 2010 [13] recommended warfarin therapy to all patients with a score of at least one which might explain this pattern. Also, aspirin was no longer recommended as an alternative to warfarin in patients with a moderate risk factor. The sudden decline in the application of aspirin may be due to the guidelines from 2010. Overall, we observed an association between new guidelines and modified use of warfarin. However, the changes seemed noticeably already one year prior to the new guidelines. Despite an increasing application, more than fifty percent of the incident AF patients did not follow the current guidelines.

In previous studies, a higher use of warfarin has been reported over the years, including a recent Danish study [14]. Shroff et al. [15] investigated the use of warfarin between 1992 and 2010 in AF patients above 64 years using the American Medicare database. In this period they observed an increased use changing from 29% to 63% in males. Warfarin usage was determined relative to three or more prothrombin time claims during the first year of AF. Pilote et al. [16] also observed an increased usage between 1998 and 2006 (51.0% to 64.5%) in incident AF patients. A Finnish study [17] examined 2,746 patients and concluded that 50.3% of all prevalent AF patients with a CHA_2_DS_2_-VASc score above zero were in therapeutic level between 2010 and 2012. Some of the big difference in warfarin usage may be due to different populations (subgroups vs. nationwide). However, the subgroups may be representative, indicating a lower use of warfarin in the Danish population.

### Thromboembolism

Events of thromboembolism had a declining trend in both warfarin and non-warfarin treated patients. This has previously been observed [15,18] and may be a result of improved lifestyle and treatment. Yet, the warfarin treated patients had a sharper decline. Better management of warfarin therapy may be a reason. To obtain a protective effect of warfarin, patients had to be in the therapeutic level. Therefore, more time in the therapeutic level may have improved the event rate. This would explain why the greatest reduction was in patients with the highest risk (CHA_2_DS_2_-VASc score >1) since they had the greatest benefit of warfarin [19]. It may be hypothesized that the improved safety regimens like clinics specialized in warfarin therapy might have worked. At first glance, the sudden increase in 2007 might be explained by a less strict use of warfarin in the guidelines from 2006 (S1 Table). As previously described, a single moderate risk factor did not necessarily trigger warfarin therapy (aspirin was an alternative). However, use of warfarin had been decreasing since 2005, and an equal increase in the use of aspirin was not observed. Further, the increase in TE was observed in all three CHA_2_DS_2_-VASc score groups. The slightly higher event rate in the warfarin treated patients with a CHA_2_DS_2_-VASc score of 1 (relative to non-warfarin treated) may be caused by the differences in age and comorbidities, Table 1.

Shroff et al. [15] also observed a declining stroke rate from 1992 to 2010 in prevalent AF patients. In contrast, Pilote et al. [16]did not observe this declining pattern in incident AF patients between 2000 and 2005. They studied stroke and transitory ischemic attack in the first year of AF and observed an event rate of 3.8% in 2000 and 3.5% in 2005. In this period we observed a decline of thromboembolic events (stroke, transitory ischemic attack, and peripheral arterial thromboembolism). In 2000 we observed a rate of 3.8% and 3.1% in 2005.

### Bleeding

The overall bleeding rate increased between 1996 and 2006. This coincides with an increased use of warfarin in this period. Yet, a very similar trend was observed between warfarin and non-warfarin treated patients having a CHA_2_DS_2_-VASc score above 1. The higher event rate may have been driven by the increasing risk of bleeding (ie., increasing HAS-BLED score) including the growing use of aspirin and NSAID. In 2011 use of aspirin and NSAID decreased in both the warfarin and non-warfarin treated patients, Fig 1. This may have contributed to the sudden decrease (relative to the trendline) in 2011.

Pilote et al. [16] studied the bleeding rate in the first year of AF. They observed an increased rate from 4.8% in 2000 to 6.1% in 2005 together with an improved use of warfarin. The greater use of warfarin might have caused the increasing event rate. Similarly, we observed an increasing trend. However, the bleeding rate was lower (3.7% in 2000 and 4.5% in 2005) which may be explained by the lower application of warfarin (31% in 2000 and 39% in 2005).

## Limitations

Prothrombin time was not included in the HAS-BLED risk score due to missing clinical data. Data on several clinical variables such as blood pressure, blood biochemistry, and echocardiographic indices was not available. Therefore, hypertension was estimated by a validated proxy based on combinations of antihypertensive medications. Information of warfarin, clopidogrel and aspirin were established by prescriptions up to 90 days prior to the index day (30 days grace period + 60 days prior to grace period). If patients changed status after the index day, this would not be registered. Unmeasured confounding might have affected the results, including e.g. a possibly differential registration of comorbidities in the registries over time, or a changing referral pattern to the hospitals. We are, however, unaware of such changes or alternation in the AF diagnostics. Because of a possible change in referral patterns, we choose to include both in- and out-hospital visits plus emergency-room visits.

## Conclusion

This study consolidated the interesting trend of decreasing TE in both treated and non-treated patients. Particularly warfarin treated patients with a CHA_2_DS_2_-VASc score above 1 had fewer events of TE. Over time, more patients having a higher risk of bleeding where using warfarin. Bleeding–an adverse effect of warfarin–increased in both warfarin and non-warfarin treated patients but did not increase considerable more in the warfarin treated patients compared to the non-warfarin treated. Still, anticoagulants therapy was only initiated in 41% of incident AF patients in 2011. We may have become better at warfarin therapy, but challenges remain with regards to the rather low initiation.

## Supporting information

S1 Definitions Non-valvular atrial fibrillation. (DOCX)

S2 Definitions Comorbidity. (DOCX)

S3 Definitions Concomitant medical therapy. (DOCX)

S4 Definitions Outcome. (DOCX)

S1 Fig Flowchart. Flowchart of the exclusions.
(DOCX)

S2 Fig Temporal trends. (DOCX)

S3 Fig Length of grace period and the effect on warfarin estimate. (DOCX)

S4 Fig Length of grace period and the effect on TE rate. (DOCX)

S5 Fig Length of grace period and the effect on bleeding rate. (DOCX)

S6 Fig Temporal trends of TE, bleeding and warfarin not excluding patients with multiple antithrombotic drug use during the grace period. (DOCX)

S1 Table Definition of risk factors according to ACC/AHA/ESC 2006 Guidelines. (DOCX)
