# Supplementary material for: Trends in warfarin use and its associations with thromboembolic and bleeding rates in a population with atrial fibrillation between 1996 and 2011
Source: PLoS One. 2018 Mar 16;13(3):e0194295. doi: 10.1371/journal.pone.0194295 (PMC5856343; doi:10.1371/journal.pone.0194295)
Supplement: S1 Definitions — (DOCX) [file pone.0194295.s001.docx]

**S1 Definitions. Non-valvular atrial fibrillation**

Present of ICD8: 42793, 42794

ICD10: DI48

Absence of ICD8: 4240, 4241, 39500-39502, 39508-39509, 39600-39604, 39608-39609

ICD10: DZ952, DZ954, DI05, DI06, DI080A- DI083A,

NCSP: KFKD, KFKH, KFMD, KFMH, KFGE, KFJF

ICD8: 8th revision of the International Classification of Diseases system

ICD10: 10th revision of the International Classification of Diseases system

NCSP: The Nordic Medical Statistics Committees Classification of Surgical Procedures
