# Supplementary material for: Trends in warfarin use and its associations with thromboembolic and bleeding rates in a population with atrial fibrillation between 1996 and 2011
Source: PLoS One. 2018 Mar 16;13(3):e0194295. doi: 10.1371/journal.pone.0194295 (PMC5856343; doi:10.1371/journal.pone.0194295)
Supplement: S2 Definitions — (DOCX) [file pone.0194295.s002.docx]

**S2 Definitions. Comorbidity**

Alcohol abuse [1]

Defined from diagnosis and adverse alcohol consumption reported during hospitalization

ICD8: 291, 303, N979-N980

ICD10: E244, E52, F10, G312, G621, G721, I426, K292, K70, K860, L278A, O354, T51, Z714, Z721

ATC:N07BB

Diabetes mellitus [1]

Defined from medical therapy: Glucose-lowering medication (ATC: A10).

ICD8: 8th revision of the International Classification of Diseases system

ICD10: 10th revision of the International Classification of Diseases system

References:

[1] J. B. Olesen, G. Y. H. Lip, A.-L. Kamper, K. Hommel, L. Køber, D. a Lane, J. Lindhardsen, G. H. Gislason, and C. Torp-Pedersen, “Stroke and bleeding in atrial fibrillation with chronic kidney disease.,” N. Engl. J. Med., vol. 367, no. 7, pp. 625–35, Aug. 2012.
