# Supplementary material for: Trends in warfarin use and its associations with thromboembolic and bleeding rates in a population with atrial fibrillation between 1996 and 2011
Source: PLoS One. 2018 Mar 16;13(3):e0194295. doi: 10.1371/journal.pone.0194295 (PMC5856343; doi:10.1371/journal.pone.0194295)
Supplement: S3 Definitions — (DOCX) [file pone.0194295.s003.docx]

**S3 Definitions. Concomitant medical therapy**

Prescriptions within 90 days prior to the index date were identified and classified as concomitant medical therapy; renin-angiotensin system inhibitors (ATC code C09A), B-blockers (ACT code C07), calcium channel blockers (ATC code C08), digoxin (ATC code C01A), amiodarone (ATC code C01BD01), and class 1C antiarrhythmics (ATC code C01BC), statins (ATC code C10AA), nonsteroidal anti-inflammatory drugs (ATC code M01A), and proton pump inhibitors (ATC code A02BC).

ICD8: 8th revision of the International Classification of Diseases system

ICD10: 10th revision of the International Classification of Diseases system
