# Supplementary material for: Trends in warfarin use and its associations with thromboembolic and bleeding rates in a population with atrial fibrillation between 1996 and 2011
Source: PLoS One. 2018 Mar 16;13(3):e0194295. doi: 10.1371/journal.pone.0194295 (PMC5856343; doi:10.1371/journal.pone.0194295)
Supplement: S4 Definitions — (DOCX) [file pone.0194295.s004.docx]

**S4 Definitions. Outcome**

Bleeding

Intracranial bleeding, Gastrointestinal bleeding, Urinary/renal bleeding, Airway bleeding, Ocular bleeding, Retroperitoneal bleeding, Intraspinal bleeding, Pericardial bleeding, Anaemia

ICD-8: 43008-43009,43099-43101, 43108-43109, 43190, 43198-43199, 85200-85202,85210-85212,85290-85292, 45600-45601, 53098, 53190, 53192, 53195, 53290, 53390, 53490, 56915, 25590, 37700-37701, 28001

ICD-10: I60-I62, S064-S066, K250, K252, K254, K256, K260, K262, K264, K266-K625, K270, K272, K274, K276, K280, K282, K284, K286, N02, R31, J942, K228F, K298A, K920-K922, DK638B, DK638C, DK661, DK838F, DK868G, I850, I864A, R04, H313, H356, H431, H450, H052A, DS368D, G951A, I312, DD62, DD500

Thromboembolism

Iskæmiske, Transient ischemic attack

Ischemic, Haemorrhagic,

ICD-8: 433-435, 43601, 43690

ICD-10: I63, I64, G459, G458, I74

ICD8: 8th revision of the International Classification of Diseases system

ICD10: 10th revision of the International Classification of Diseases system
