# Supplementary material for: Trends in warfarin use and its associations with thromboembolic and bleeding rates in a population with atrial fibrillation between 1996 and 2011
Source: PLoS One. 2018 Mar 16;13(3):e0194295. doi: 10.1371/journal.pone.0194295 (PMC5856343; doi:10.1371/journal.pone.0194295)
Supplement: S1 Fig — Flowchart of the exclusions. (DOCX) [file pone.0194295.s005.docx]

**S1 Fig. Flowchart.** Flowchart of the exclusions. TE indicates thromboembolism.
