# Supplementary material for: Trends in warfarin use and its associations with thromboembolic and bleeding rates in a population with atrial fibrillation between 1996 and 2011
Source: PLoS One. 2018 Mar 16;13(3):e0194295. doi: 10.1371/journal.pone.0194295 (PMC5856343; doi:10.1371/journal.pone.0194295)
Supplement: S2 Fig — (DOCX) [file pone.0194295.s006.docx]

**S2 Fig. Temporal trends.** Temporal trends of the variables included in the HAS-BLED and CHA_2_DS_2_VASc scores stratified relative to warfarin and non- warfarin therapy. Year of AF diagnosis indicates the year AF was diagnosed.
