# Supplementary material for: Trends in warfarin use and its associations with thromboembolic and bleeding rates in a population with atrial fibrillation between 1996 and 2011
Source: PLoS One. 2018 Mar 16;13(3):e0194295. doi: 10.1371/journal.pone.0194295 (PMC5856343; doi:10.1371/journal.pone.0194295)
Supplement: S4 Fig — (DOCX) [file pone.0194295.s008.docx]

**S4 Fig. Length of grace period and the effect on TE rate.** Comparing the effect of a 7 and a 30 days grace periode regarding the number of thromboembolic event rate. Te indicates thromboembolic event.
