# Supplementary material for: Trends in warfarin use and its associations with thromboembolic and bleeding rates in a population with atrial fibrillation between 1996 and 2011
Source: PLoS One. 2018 Mar 16;13(3):e0194295. doi: 10.1371/journal.pone.0194295 (PMC5856343; doi:10.1371/journal.pone.0194295)
Supplement: S6 Fig — (DOCX) [file pone.0194295.s010.docx]

**S6 Fig. Temporal trends of TE, bleeding and warfarin not excluding patients with multiple antithrombotic drug use during the grace period.** Left axis illustrates the percentage of AF patients having an event within the first year. Right axis illustrates the percentage of patients initiating warfarin. TE indicates events of thromboembolism; bleeding, events of bleedings; warfarin, initiation of warfarin; and Year of AF diagnosis, the year the patients got their AF diagnosis.
