# Supplementary material for: Trends in warfarin use and its associations with thromboembolic and bleeding rates in a population with atrial fibrillation between 1996 and 2011
Source: PLoS One. 2018 Mar 16;13(3):e0194295. doi: 10.1371/journal.pone.0194295 (PMC5856343; doi:10.1371/journal.pone.0194295)
Supplement: S1 Table — (DOCX) [file pone.0194295.s011.docx]

**S1 Table. Definition of risk factors according to ACC/AHA/ESC 2006 Guidelines.**

| **Less Validated or Weaker Risk Factors** | **Moderate-Risk Factors** | **High-Risk Factors** |
| --- | --- | --- |
| Female gender | Age greater than or equal to 75 year | Previous stroke, TIA or embolism |
| Age 65 to 74 year | Hypertension | Mitral stenosis |
| Coronary artery disease | Heart failure | Prosthetic heart valve |
| Thyrotoxicosis | LV ejection fraction 35% or less |  |
|  | Diabetes mellitus |  |
